# Prediction of Compressive Strength of Biomass–Humic Acid Limonite Pellets Using Artificial Neural Network Model

**DOI:** 10.3390/ma16145184

**Published:** 2023-07-24

**Authors:** Haoli Yan, Xiaolei Zhou, Lei Gao, Haoyu Fang, Yunpeng Wang, Haohang Ji, Shangrui Liu

**Affiliations:** Faculty of Metallurgical and Energy Engineering, Kunming University of Science and Technology, Kunming 650093, China; ximengfunny@163.com (H.Y.); fanghaoyuya1@163.com (H.F.); wangypeng1997@163.com (Y.W.); jhhkust2022@163.com (H.J.); lsr190608@163.com (S.L.)

**Keywords:** neural network, limonite, pelletizing, compressive strength, organic binder

## Abstract

Due to the detrimental impact of steel industry emissions on the environment, countries worldwide prioritize green development. Replacing sintered iron ore with pellets holds promise for emission reduction and environmental protection. As high-grade iron ore resources decline, research on limonite pellet technology becomes crucial. However, pellets undergo rigorous mechanical actions during production and use. This study prepared a series of limonite pellet samples with varying ratios and measured their compressive strength. The influence of humic acid on the compressive strength of green and indurated pellets was explored. The results indicate that humic acid enhances the strength of green pellets but reduces that of indurated limonite pellets, which exhibit lower compressive strength compared to bentonite-based pellets. Furthermore, artificial neural networks (ANN) predicted the compressive strength of humic acid and bentonite-based pellets, establishing the relationship between input variables (binder content, pellet diameter, and weight) and output response (compressive strength). Integrating pellet technology and machine learning drives limonite pellet advancement, contributing to emission reduction and environmental preservation.

## 1. Introduction

Many countries worldwide are implementing green development initiatives to reduce carbon emissions [1,2]. The steel industry, as a heavy industry, generates a significant amount of exhaust gases, wastewater, and solid waste during the production process, leading to severe environmental pollution [3,4]. In comparison to sintered ore, pelletized ore has higher iron content, fewer harmful elements, and good reducibility, which results in increased production, reduced coke consumption, cost savings, optimized burden structure, and improved economic benefits in blast furnace smelting. Therefore, it is necessary to continuously increase the proportion of pelletized ore in the ironmaking process [5,6].

To date, extensive research has been conducted on the preparation of high-grade ore pellets [7]. However, with the rapid development of the steel industry, the output of rich ores has decreased, while the extraction of low-grade ores has increased [8,9,10,11,12]. The technology related to pellet production using low-grade ores is still not mature, necessitating further research on low-grade ore pellets. Limonite, characterized by low levels of harmful elements, is an example of low-grade iron ore. However, low-grade ores often contain numerous impurities, resulting in lower pellet strength when using limonite. Moreover, pelletized ores undergo multiple handling, transportation, stacking, and movement processes before and after entering the blast furnace, experiencing various harsh mechanical forces such as collisions, impacts, compression, and friction [13]. These mechanical forces can cause the breakage of some pellets, leading to the generation of fines and affecting furnace operation and production indicators. To address these challenges, the addition of binders to the pellets can fill the gaps and cracks between ore particles, bonding them together to increase the density and mechanical strength of the pellets [14].

Bentonite, as the main binder for pellets, cannot undergo thermal decomposition during the smelting process and mostly remains in the pellets, leading to a decrease in the iron grade of the pelletized ore [15]. Qiu et al. [16] utilized organic binders to prepare pelletized ore and found that, compared to pellets prepared using bentonite, the resulting pellets had a higher iron grade and lower impurity content [17]. Humic acid, as an organic binder and a biomass-derived substance, exhibits strong adsorption capacity on the surface of iron ore, thereby enhancing the strength of green pellets [18,19]. Currently, there is limited research on the preparation of limonite pellets using humic acid as a binder, necessitating further investigation into humic acid-based limonite pellets. However, most studies on pellet compressive strength have relied on single-factor analysis or orthogonal experiments to qualitatively describe the influencing factors of various parameters, without considering the interactions among them [20]. Due to the limited number of experiments, the predictability is relatively poor [21]. Artificial neural networks (ANNs) offer a promising solution for predicting material properties and have been widely used in this context [22]. ANNs are often referred to as “black-box” models because they can make accurate predictions based on training data without providing any physical explanation behind the phenomena [23,24].

Numerous studies have harnessed the power of Artificial Neural Networks (ANN) in diverse applications within the field of materials science and metallurgy. Chagas et al. [25] applied ANN to assess the sensitivity of variables in pellet bed formation, aiding in the generation of green pellets with reduced fuel and energy consumption and improved final quality. Dwarapudi et al. [26] developed an ANN model to predict the cold compressive strength (CCS) of pellets in a straight grate furnace. By considering variations in bentonite, alkalinity, FeO, and green pellet moisture, the model successfully predicted CCS with an error margin of less than 3%. Klippel et al. [27] introduced an early detection system for slope instability risks based on iron ore images, utilizing edge artificial intelligence. The field test results demonstrated an accuracy rate of 91% and a recall rate of 96%, highlighting the feasibility of employing deep learning for detecting iron ore types and preventing slope instability risks. Fan et al. [28] investigated the main factors affecting sintering quality, such as humidity, fuel ratio, sintering speed, and sintering drum strength. Utilizing backpropagation ANN, prediction models were constructed and applied in sintering pot experiments to optimize humidity and fuel ratio, ultimately improving sintering drum strength. Golmohammadi et al. [29] developed a Quantitative Structure–Property Relationship (QSPR) using Partial Least Squares (PLS) and ANN to predict the precipitation of trivalent iron during bioleaching. The neural network model exhibited reliable and accurate predictive capabilities during the bioleaching process. Li Guo et al. [30] proposed a novel method to estimate ore feed load directly from images of ore pellets using deep learning models. The introduction of a weakly supervised learning method and a two-stage model training algorithm allowed competitive model performance and real-time estimation of ore feed load in the grinding process optimization. Li et al. [31] developed an intelligent system named the Group Method of Data Handling (GMDH) for predicting iron ore prices. Compared to other techniques, the GMDH technique exhibited superior accuracy with a variance accounted for (VAF) value of 97.89%. Wang et al. [32] successfully implemented a hybrid ensemble model combining Extreme Learning Machine (ELM) with an improved AdaBoost. The RT algorithm is used to solve regression problems in sintering processes. This approach led to significant improvements in energy efficiency and sintering quality through the analysis of high-priority factors. Yachun Mao et al. [33] introduced a detection method for the magnetic properties of limonite using the improved Particle Swarm Optimization–Enhanced Extreme Learning Machine (IPSO–ELM) algorithm and spectroscopy. The IPSO–ELM predictive model exhibited excellent performance and generalization capability compared to ELM and PSO–ELM predictive models. Yanwei Yang et al. [34] successfully integrated laser-induced breakdown spectroscopy (LIBS) and machine learning for rapid and precise classification of iron ore, providing a novel method for iron ore selection in the metallurgical industry. Tunckaya et al. [35] utilized ANN, Multiple Linear Regression (MLR), and auto-regressive integrated moving average (ARIMA) models to predict and track the flame temperature of a blast furnace. The computational results demonstrated satisfactory performance in the selected performance indicators, including regression coefficients and root mean square errors.

Previous studies on pellet compressive strength mostly relied on single-factor analysis or orthogonal experiments to qualitatively describe the influencing factors, resulting in limited predictability. Therefore, this study introduces an innovative approach by applying an Artificial Neural Network (ANN) for data analysis and optimization, offering a novel solution for the preparation and sintering process of limonite pellets. This contributes to enhanced process efficiency, reduced production costs, and mitigated environmental impact, providing significant practical application value.

## 2. Experiment

### 2.1. Experimental Material

The limonite used in this study was obtained from a factory in Yunnan Province, China. The limonite exhibits a rough surface, porous texture, and low levels of harmful elements. Its chemical composition is presented in Table 1, showing a high content of total iron (TFe) at 54.67 wt.%, along with 4.04 wt.% of SiO_2_ and 3.47 wt.% of MnO. Notably, this limonite possesses strong adhesive properties and high water absorption capacity.

### 2.2. Experimental Procedure

**Figure 1:**
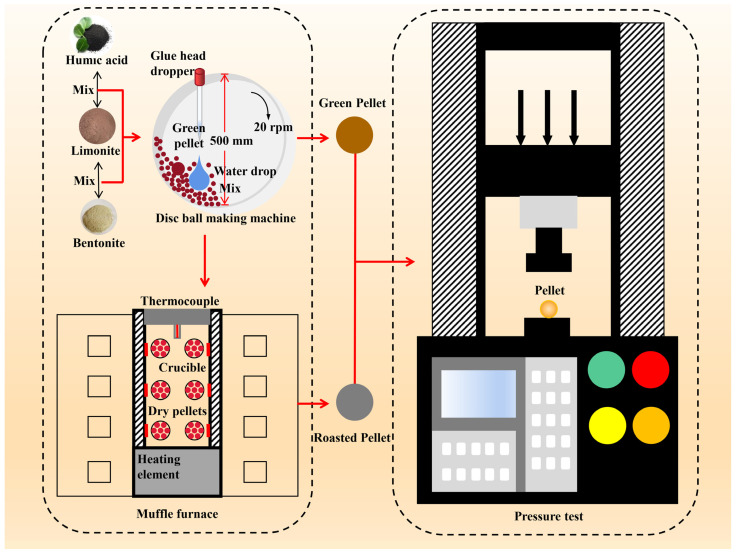
Experimental flow.

**Figure 2:**
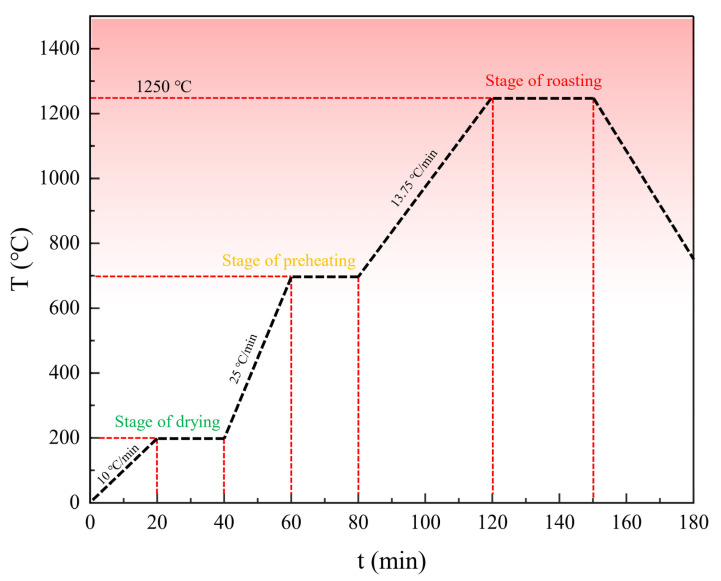
Pellet roasting temperature diagram.

The experimental procedure is illustrated in Figure 1:

(1) Pellet Manufacturing: 200 g of experimental material, limonite powder, is weighed. Binders in different proportions (0.4 wt.%, 0.8 wt.%, 1.2 wt.%, 1.6 wt.%, and 2.0 wt.%) are added, and the materials are thoroughly mixed using the multiple folding and stirring method. The balling machine is started, and approximately 2/3 of the mixed material is added to the machine. Water is slowly dripped onto the surface of the mixture to form pellets with a diameter of approximately 3 mm over a period of 3 min. Every 3 min, a mist of water is sprayed onto the surface of the mother pellets, and material is added to the wetted surface to allow the pellets to continuously roll and grow. The process is generally controlled within 12 min to achieve pellets of the desired size (9–16 mm). After stopping the addition of water and material, the pellets are allowed to continue rotating in the balling machine for approximately 2 min to achieve compaction. The pellets are removed using a small scoop. The pellets are separated using a vernier caliper, and the pellets with a diameter of 9–16 mm are considered qualified products, while the remaining pellets are deemed unqualified.

(2) Pellet Roasting: The manufactured pellets are transferred in batches into crucibles. The crucibles containing the pellets are placed into a muffle furnace for roasting. The roasting temperature is set as follows, as shown in Figure 2: Ramp up to 200 °C in 20 min and hold at 200 °C for 20 min; Ramp up to 700 °C in 20 min and hold at 700 °C for 20 min; ramp up to 1250 °C in 40 min and hold at 1250 °C for 25 min. Subsequently, the pellets are gradually cooled to room temperature and removed from the furnace.

(3) Measurement and Testing: The weight of both the raw pellets and the roasted pellets is measured using an electronic balance. The compressive strength of the raw pellets and the roasted pellets is measured using a YAW-100C testing machine. Pellets with a diameter of 9–16 mm are selected using a vernier caliper. Following the guidelines of YB/T 4848-2020, “Physical Test Methods for Roasted Pellets,” the average compressive strength of the limonite raw pellets and the roasted pellets is recorded.

## 3. Results and Discussion

### 3.1. Effect of Humic Acid on Compressive Strength of Limonite Green Pellets

**Figure 3:**
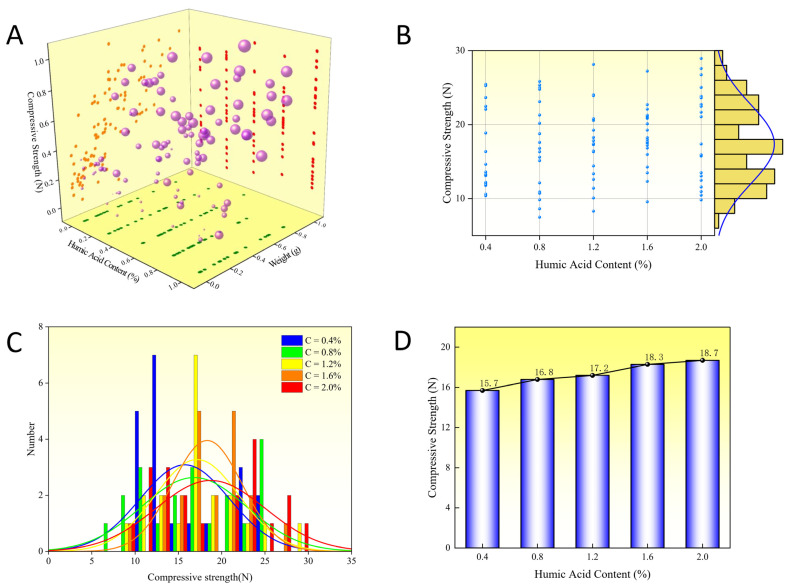
Effect of humic acid on compressive strength of limonite green pellets. (**A**) Stereo scatter data. (**B**) Planar scatter data. (**C**) Normal distribution of data. (**D**) The average of the scatter data.

As shown in Figure 3A, the influence of humic acid content, pellet weight, and diameter on the compressive strength of limonite pellets is illustrated. The diameter of the pellets is represented by the size of the purple spheres, and the data for the four parameters have been normalized. The compressive strength of the pellets increases with the weight, and larger pellets are predominantly located at the top, while smaller pellets are mainly located at the bottom. The variation in compressive strength with increasing humic acid content is not significant and is primarily related to the diameter and weight. Figure 3B,C present the scatter plot and normal distribution plot illustrating the effect of different proportions of humic acid on the compressive strength of the pellets. The humic acid content has a positive correlation with the compressive strength of the pellets. The impact of different proportions of humic acid on the compressive strength of the pellets is depicted in Figure 3D; as the proportion of humic acid in the pellet increases from 0.4% to 2.0%, the average compressive strength of the pellets rises and reaches its maximum value of 18.7 N when the humic acid content reaches 2.0%.

When the mineral powder is wetted by water during the rolling process, it forms pellets of a certain size and imparts them with a certain strength through the combined action of capillary force, molecular attraction, and frictional force. The pellet size, moisture content, mechanical strength, and thermal stability of the pellets influence the subsequent roasting operation and are related to the yield and quality of the pelletized ore.

**Figure 4:**
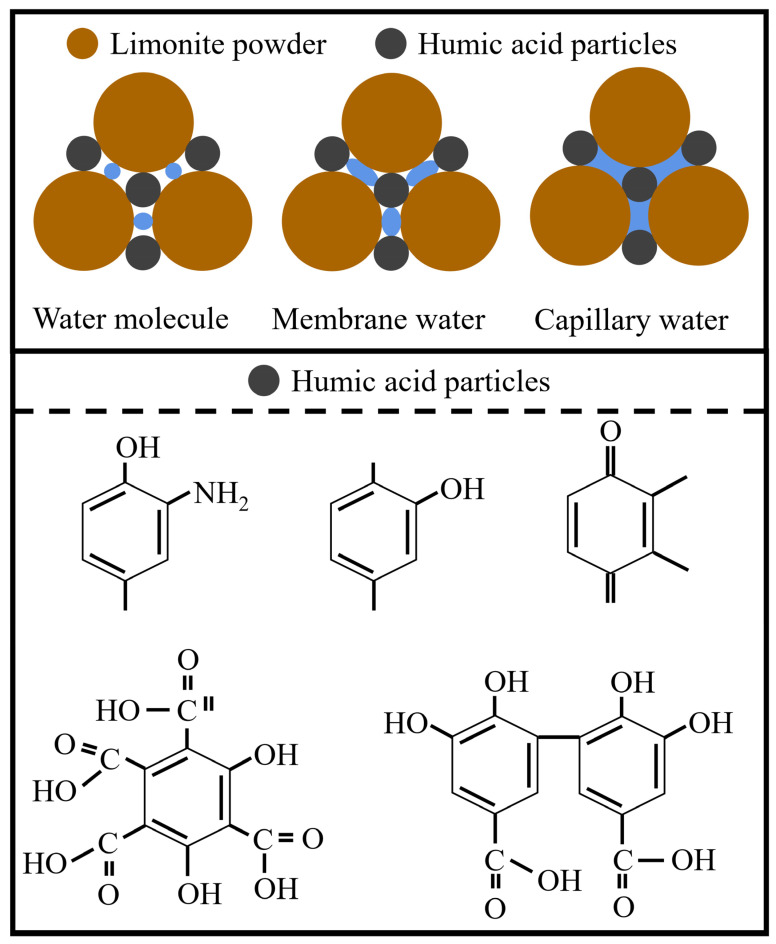
Pellet forming principle.

Dry mineral powders generally exhibit hydrophilic properties. As shown in Figure 4, under the molecular forces on the particle surface, water molecules are adsorbed onto the surface of the particles. Due to the action of molecular attraction, a thin film of water is formed outside the adsorbed water layer. The inner layer of the thin film water, which is closer to the particles, experiences stronger cohesive forces and is called bound water. It, together with the adsorbed water, is referred to as maximum dividable water, which enables the powder to be shaped but still lacks plasticity. The outer layer of the thin film water is closer to free water and can undergo plastic deformation under external forces. When the mineral powder is wetted by water, and the amount of water exceeds that of the thin film water, capillary water appears between the particles, initially in a contact state, connecting the particles. Further wetting leads to a honeycomb state, where the particles come closer together under the influence of water surface tension and external forces. Continued wetting results in the saturation state of capillary water, generating the strongest capillary forces between the particles.

As an organic binder, humic acid not only improves the grade of metallic pellets but also accelerates reduction where possible. It exhibits high particle size at room temperature and high bonding strength after drying. The addition of a small amount of organic binder significantly enhances the compressive strength of the pellets. This is because humic acid contains a considerable amount of carboxylate ions, hydroxyl groups, and other oxygen-containing functional groups, indicating its strong hydrophilicity. Moreover, carboxyl groups can form complex or chelation reactions with metal ions and metal hydroxides, facilitating chemical adsorption between humic acid and the surface of iron ore particles, resulting in strong binding forces and improved pellet strength.

### 3.2. Effect of Humic Acid on Compressive Strength of Limonite Roasted Pellets

**Figure 5:**
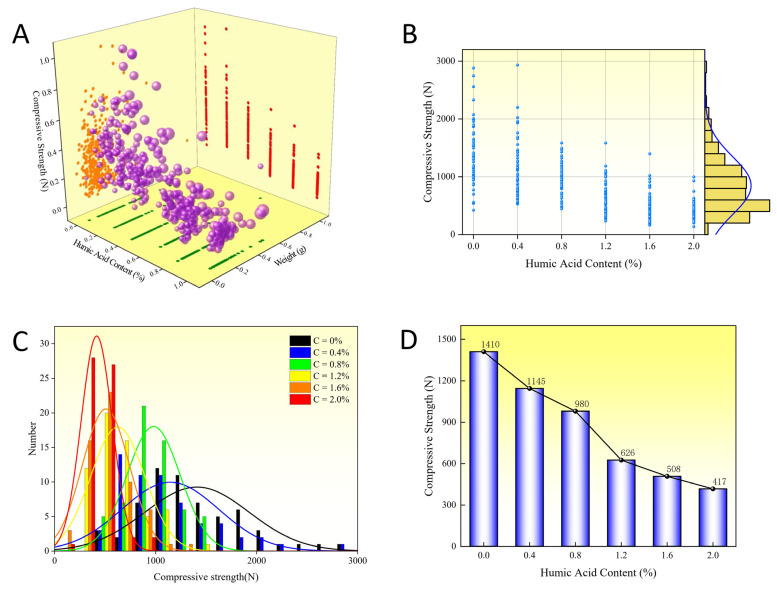
Effect of humic acid on compressive strength of limonite roasted pellets. (**A**) Stereo scatter data. (**B**) Planar scatter data. (**C**) Normal distribution of data. (**D**) The average of the scatter data.

As shown in Figure 5A, the influence of humic acid content, roasted pellet weight, and diameter on the compressive strength of roasted limonite pellets is depicted. The size of the pellets is represented by the size of purple spheres, and the data for the four parameters have been normalized. The compressive strength of the pellets increases with increasing weight, with larger pellets mainly distributed towards the upper end and smaller pellets towards the lower end. With the increase in humic acid content, the compressive strength of the roasted pellets decreases. Figure 5B,C presents scatter plots and normal distribution plots illustrating the effect of different proportions of humic acid on the compressive strength of the roasted pellets. The humic acid content has a significant impact on the compressive strength of the roasted pellets, showing a negative correlation. The effect of different proportions of humic acid on the compressive strength of the roasted pellets is illustrated in Figure 5D; when the proportion of humic acid in the pellet ore increases from 0.4% to 2.0%, the average compressive strength of the roasted pellets decreases, reaching a minimum value of 417 N at a humic acid content of 2.0%.

The consolidation of pellet ore primarily relies on solid-state reactions. This includes the solid-state diffusion consolidation of individual-phase particles at high temperatures and the formation of compounds or solid solutions through solid diffusion in multicomponent systems. These processes generally occur below their melting temperatures without the generation of a liquid phase, enabling the consolidation of pellet ore with sufficient strength. It is important to note that the complete exclusion of a liquid phase is not necessary for pellet ore consolidation, although the presence of a liquid phase is minimal. From a consolidation principal perspective, pellet ore consolidation can occur without a liquid phase. Under microscopic observation, the liquid phase in pellet ore typically does not exceed 5%, although self-melting pellet ore may contain a higher amount of liquid phase.

As the roasting temperature increases, various physical and chemical reactions within the pellet ore are accelerated, leading to increased particle diffusion and contact area. The interparticle pores gradually become rounded and reduced. At high temperatures, two processes occur within the pellet: recrystallization and grain growth. These processes influence the microstructure of the pellet, including grain size and pore distribution, resulting in the formation of a dense sphere and improved strength of the final product. The compressive strength of the roasted pellet is significantly higher than that of the raw pellet.

When the content of humic acid in the pellet increases, the compressive strength of the roasted pellet decreases. For humic acid, excessively high solution viscosity has a negative impact on the compressive strength of the roasted pellet. A high concentration of humic acid leads to increased solution viscosity, inhibiting particle diffusion within the pellet and reducing the contact area. This hampers various reactions and also suppresses the recrystallization process within the pellet, thus impeding pellet ore consolidation. Additionally, as an organic binder, humic acid is prone to volatilization during the pellet roasting process, leaving behind voids within the pellet, further reducing its compressive strength.

### 3.3. Effect of Bentonite on Compressive Strength of Limonite Roasted Pellets

**Figure 6:**
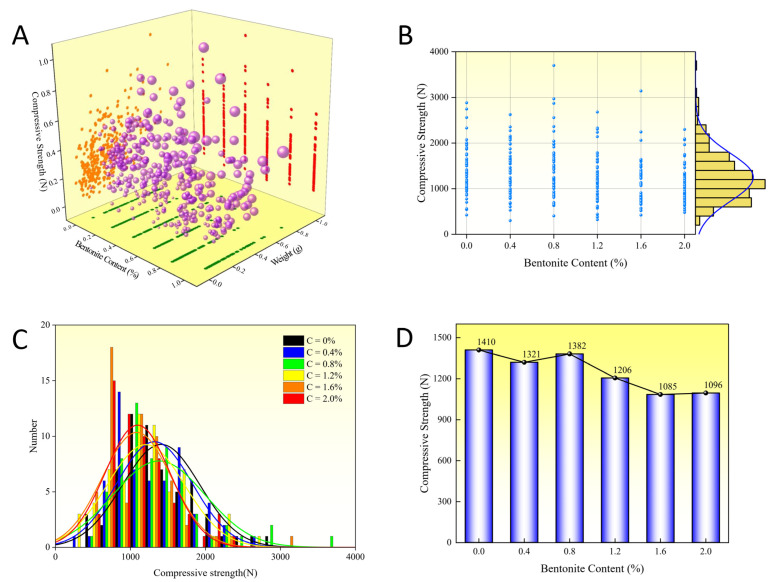
Effect of bentonite on compressive strength of limonite roasted pellets. (**A**) Stereo scatter data. (**B**) Planar scatter data. (**C**) Normal distribution of data. (**D**) The average of the scatter data.

As shown in Figure 6A, the influence of bentonite content, roasting pellet weight, and diameter on the compressive strength of roasted limonite pellets is depicted. The size of the pellets is represented by the diameter of purple spheres, and the data for these four parameters have been normalized. The compressive strength of the roasted pellets increases with an increase in weight, with larger pellets predominantly distributed in the upper range and smaller pellets in the lower range. On the other hand, as the bentonite content increases, there is a downward trend observed in the compressive strength of the roasted pellets. Figure 6B,C illustrates the scatter plot and normal distribution plot, respectively, demonstrating the impact of different proportions of bentonite on the compressive strength of roasted pellets. The bentonite content indeed has an effect on the compressive strength of the roasted pellets, showing an overall negative correlation. Figure 6D presents the effects of different proportions of bentonite on the compressive strength of roasted pellets. It can be observed that as the bentonite content in the pellet ore increases from 0.4% to 2.0%, there is an overall decreasing trend in the average compressive strength of the roasted pellets. The minimum value of compressive strength is reached at a bentonite content of 1.6%, measuring 1085 N.

**Figure 7:**
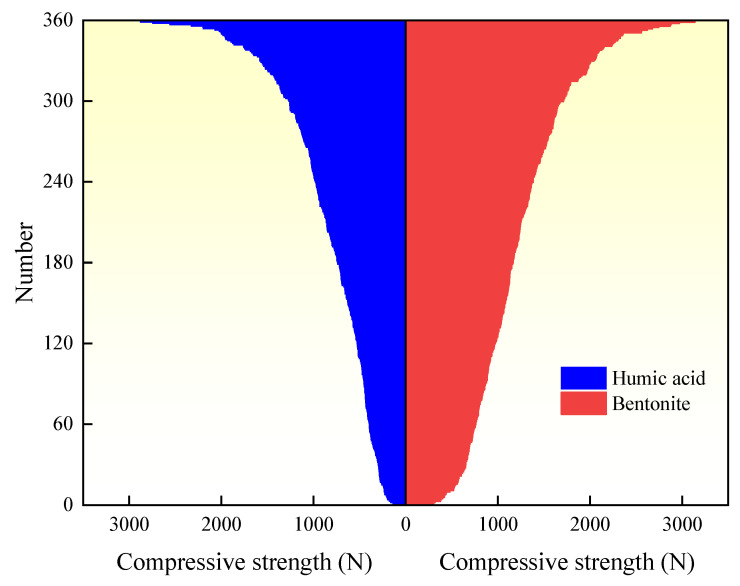
Comparison between humic acid pellets and bentonite pellets.

As shown in Figure 7, a comparative illustration of the influence of humic acid and bentonite on the compressive strength of roasted limonite pellets is presented. The compressive strength data is arranged in descending order on both sides, with a total of 360 data points. It can be observed that the compressive strength of the pellets with bentonite is generally higher than that of the pellets with humic acid, indicating that the compressive strength of the pellets produced using humic acid as a binder is inferior to those containing bentonite after roasting. In comparison to the bentonite pellets, the particles in the humic acid pellets are more dispersed and smaller in size. To form a connected crystalline structure, it is essential for the particles to come into contact with each other, which suggests that the particle contact in the humic acid pellets is not as close as in the bentonite pellets. Additionally, the generation of a liquid phase by low-melting substances facilitates solid-state diffusion and promotes crystal growth.

The difference in particle contact between these two types of pellets is primarily attributed to the small particle size and good dispersibility of bentonite, which fills the interstices between mineral particles in a colloidal state, thereby improving the particle size distribution of the pellet raw materials and reducing the pellet porosity. On the other hand, during the preheating process, humic acid undergoes combustion and decomposition, resulting in the formation of micro-pores in the pellets. Furthermore, the mechanisms of bentonite and humic acid differ. In the pellet formation process, the main binding forces between particles are interfacial forces and capillary forces. When the voids are completely filled with liquid, capillary forces play a major role in particle bonding. Bentonite fills the interstices between particles, reducing the capillary diameter within the green pellets and increasing capillary forces. It also enhances the molecular bonding forces between particles, resulting in closer particle arrangement. On the other hand, humic acid dissolves in water, exhibiting high viscosity and forming a network structure within the pellets to bind the particles together. However, due to the high viscosity, the liquid is difficult to be expelled from the capillaries, resulting in high moisture content within the green pellets and less compact particle arrangement compared to the bentonite pellets.

### 3.4. Predicting the Compressive Strength of Indurated Pellets Using an Artificial Neural Network Model

The Artificial Neural Network (ANN) is a computational model inspired by the biological neural system. It consists of a large number of interconnected artificial neurons (neuron models), resembling the connections between neurons in the human brain. Each artificial neuron receives multiple inputs, undergoes weighted processing, and generates output through an activation function. The training process of ANN resembles human learning. By iteratively adjusting connection weights, ANN can learn and extract features from input data, enabling tasks such as pattern recognition, data classification, and function approximation. It exhibits high parallel processing capability and adaptability, capable of handling complex nonlinear relationships and large-scale datasets.

**Figure 8:**
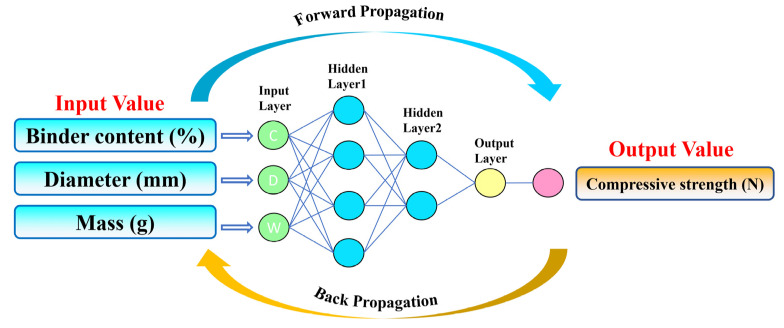
Artificial neural network model.

In this study, the experimental investigation focused on the impact of binder content, pellet diameter, and pellet weight on compressive strength. The ANN model (Figure 8) design utilized binder content, pellet diameter, and pellet weight as input layers and compressive strength as the output layer. The experimental dataset was normalized and randomized using Excel’s random function. Subsequently, the dataset was divided into two parts: a training dataset (top 80%) and a testing dataset (bottom 20%). The model’s performance was evaluated using the correlation coefficient (R^2^). The number of epochs is set to 1000.

**Figure 9:**
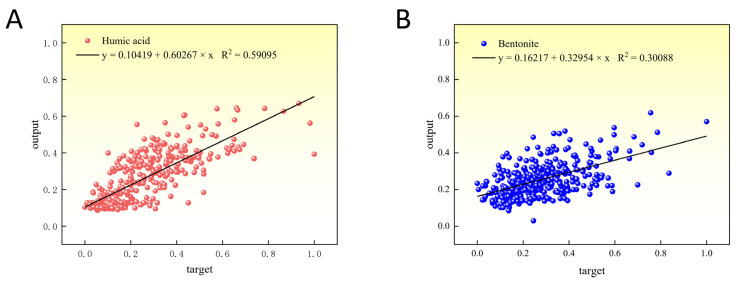
Data regression graph using artificial neural network. (**A**) Humic acid. (**B**) Bentonite.

As shown in Figure 9, a scatter plot illustrates the correlation between the target values (experimental results) and the predicted values for the compressive strength of two types of indurated pellets, namely, humic acid-based and bentonite-based pellets. Linear regression analysis was performed on the data, yielding the linear regression equations and R-squared values, which were found to be 0.59095 and 0.30088 for humic acid-based and bentonite-based pellets, respectively.

**Figure 10:**
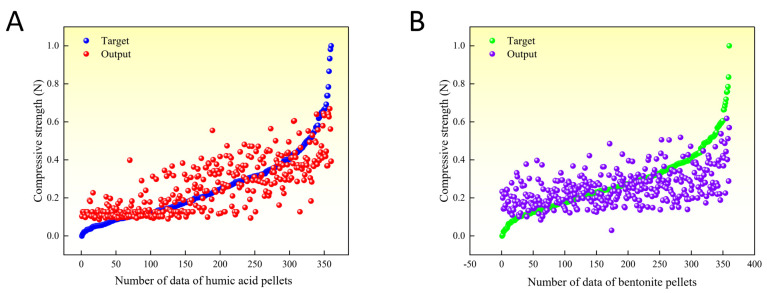
Scatter plot of target and output values. (**A**) Humic acid. (**B**) Bentonite.

Figure 10 depicts the scatter plot of target values (experimental results) versus predicted values for the compressive strength of the indurated pellets. Each target value corresponds to an output value, and all target values are arranged in ascending order. Smaller distances between target values and output values indicate higher predictive accuracy and smaller errors. The plot reveals a consistent trend among the 360 target values and their corresponding output values.

**Figure 11:**
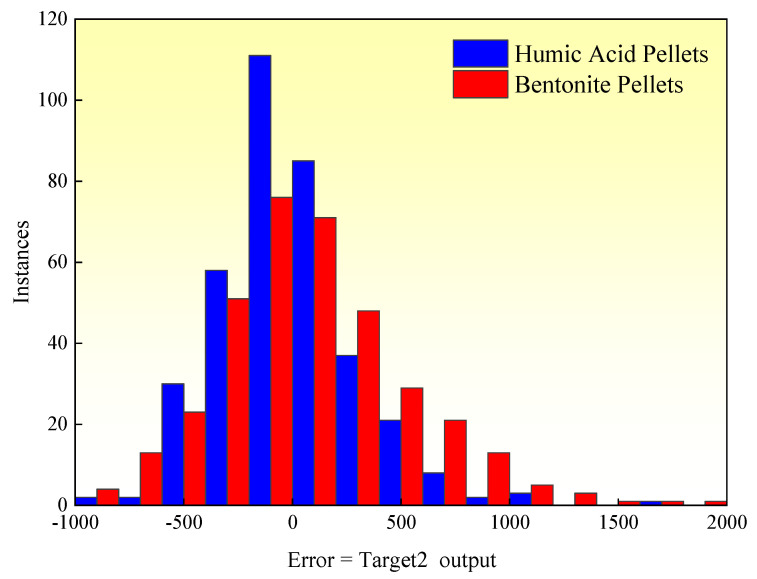
Error histogram.

Furthermore, Figure 11 displays the absolute errors (target value − output value) between the target values (experimental results) and predicted values for the compressive strength of the indurated pellets. The errors follow a normal distribution pattern centered around zero. These findings demonstrate that the artificial neural network model designed in this study provides highly accurate predictions for the compressive strength of humic acid-based indurated limonite pellets. Importantly, the model successfully establishes the relationship between input variables (binder content, pellet diameter, and pellet weight) and the output variable (compressive strength).

## 4. Conclusions

Contributing to the reduction of industrial exhaust emissions and environmental protection, this study investigates the impact of humic acid on the compressive strength of limonite pellets, aiming to elucidate the physical properties of these pellets. The results demonstrate that as the content of humic acid increases, the compressive strength of raw limonite pellets shows an upward trend, with an increase from 15.7 N/pellet to 18.7 N/pellet. Conversely, the compressive strength of roasted limonite pellets exhibits a downward trend, decreasing from 1410 N/pellet to 417 N/pellet. Similarly, with an increase in bentonite content, the compressive strength of roasted limonite pellets shows a declining trend, decreasing from 1410 N/pellet to 1096 N/pellet. The lower strength of humic acid-roasted pellets compared to bentonite-roasted pellets can be attributed to the fewer contact points between particles within the pellets, higher porosity, lower probability of particle reactions, and fewer low-melting-point substances acting as binders within the pellets. The R^2^ values for humic acid-roasted pellets and bentonite-roasted pellets are 0.59095 and 0.30088, respectively. The 360 experimental values align with the predicted values, and their absolute errors (target values–output values) exhibit a normal distribution centered around zero. These results indicate that the artificial neural network model designed in this study achieves high accuracy in predicting the compressive strength of humic acid-roasted limonite pellets.

## Figures and Tables

**Table 1:** Chemical composition of precious sand limonite (wt.%).

TFe	FeO	SiO_2_	S	MnO	TiO_2_	Pb	Zn	K_2_O	Na_2_O	Cu	V_2_O_5_	LOI
54.67	0.29	4.04	0.048	3.47	0.27	0.009	0.018	0.095	0.001	0.009	0.04	14.84

## Data Availability

Not applicable.
